# A study on the establishment of a traumatic brain injury model of tree shrews and the observation of neuroinflammatory responses

**DOI:** 10.1016/j.ibneur.2025.11.003

**Published:** 2025-11-04

**Authors:** Wen Yu, Li Yang, Xiaolina Zhang, Jiayu Zhang, Jie Zhang, Linbo Wang, Yongjie Huang, Shengxiong Hong, Linyi Chen, Haiying Wu, Jinglin Li

**Affiliations:** Kunming Medical University, Kunming, China

**Keywords:** Traumatic brain injury, Tree shrews, Magnetic resonance imaging, Neuroinflammatory response

## Abstract

**Background and objective:**

The tree shrew (Tupaia belangeri) has become an ideal model for studying various neurological diseases. However, there is no stable tree shrew model of traumatic brain injury (TBI) at present. This study aimed to establish a tree shrew TBI model and clarify the expression characteristics of inflammatory factors after brain injury, as well as the polarization characteristics of glial cells.

**Methods:**

Twelve adult female tree shrews were randomly divided into the Sham group and the TBI group, the Marmarou weight-drop impact method was used to establish a TBI model. Open field experiments were conducted after modeling to evaluate the motor ability of tree shrews on the 1st and 7th day. Moreover, magnetic resonance imaging (MRI), HE staining were performed to detect the imaging and morphology changes caused by trauma. The expression levels of inflammatory factors were detected by ELISA 7th days after TBI. Finally, immunofluorescence and Western Blot were used to detect the polarization characteristics of microglia and astrocytes.

**Results:**

Open field experiments showed that the total motor distance of tree shrews in TBI group on the 1st and 7th days were significantly reduced. MRI showed high-density shadow in the parietal brain parenchyma, surrounded by low-density edema bands. HE staining showed in the TBI group, some neurons in the hippocampal region showed morphological abnormalities and nuclear condensation, glial cell proliferation, the arrangement of neurons in the cortical area is disordered, and the hierarchical structure is damaged. ELISA assays showed that the levels of pro-inflammatory factors in TBI group were significantly increased, while the anti-inflammatory factors’ expressions decreased. Finally, Immunofluorescence staining showed that, compared with Sham group, fluorescence signals of M1 phenotype macroglia marker CD86 and M2 phenotype marker CD206 in different regions of hippocampus in TBI group were significantly enhanced at 7 days after TBI. Western Blotting showed that compared with sham group, the expression of A1 phenotype marker Serping1and A2 phenotype marker Ptx3 protein of astrocytes in TBI group was significantly up-regulated. ELISA results showed that the expression levels of C3 and S100a10 in brain tissue of tree shrews in TBI group were significantly higher than those in Sham group.

**Conclusions:**

This study successfully constructed a TBI tree shrew model and confirmed that the polarization imbalance of microglia and astrocytes may form a neurotoxic microenvironment, ultimately causing brain tissue damage.

## Introduction

Traumatic brain injury (TBI) is a common neurological disease with high morbidity, mortality and disability (Bergquist et al., 2024). Currently, specific treatment methods are lacking. Understanding the pathophysiological process of secondary brain injury is of great significance for improving the treatment rate.

Neurological injury after TBI includes primary and secondary injury. Primary injury is irreversible brain tissue damage directly caused by mechanical external forces. Secondary injury involves complex biological processes such as blood-brain barrier disruption and dysfunction (Cash & Theus, 2020), necrosis of nerve cells (Qin et al., 2021), programmed cell death, and neuroinflammatory responses (Ghaith et al., 2022). The development of studies on the pathologic mechanism of TBI depends on the damage replication of animal models at the molecular, cellular and biological levels (Lu Qing et al., 2023). Different experimental animal models have unique values, reducing data deviations and improving result reliability. Early TBI models used non-primate animals, like cats, dogs and pigs; later, rodents became mainstream due to low cost and easy operation. However, rodents differ greatly from humans in neuroanatomy, immunity and cognition, failing to simulate complex human brain structures and disorders, leading to low translation efficiency of results. Sheep and pigs are suitable for TBI research due to brain structure advantages but are limited by high costs and difficult genetic regulation.

Thus, finding an animal model with human-like brain anatomical structure, controllable economic costs, and easy operation is of great significance for TBI pathophysiological mechanism research. Tree shrews, confirmed to have the closest kinship with primates (Yao et al., 2024), are highly similar to primates and even humans in physiological anatomy, neural development, and psychological stress patterns. They have been used to construct high-resolution neuroanatomical datasets (Zhang et al., 2025) and are increasingly applied in neuroscience research with advanced tools (Savier et al., 2021). In the nervous system, their motor ability surpasses that of rodents, indicating well-developed neural systems (Lin et al., 2013). Anatomically, they have a well-developed frontal lobe like primates, with a brain/body weight ratio even higher than that of humans. In behavioral and emotional performance, tree shrews exhibit complex social interactions, stress responses, and advanced learning and memory behaviors, enabling evaluation of cognitive and behavioral impairments after TBI. Immunologically, they have immune response characteristics closer to humans such as the timeline and mechanism of pro-inflammatory factor production in neuroinflammation, better simulating the inflammatory pathological process of human TBI. Their adrenal glands also show human-like features (Jiang et al., 2024), further supporting their suitability as human disease models.

These developmental characteristics make tree shrews an ideal model for human neurological diseases (Wang et al., 2019). At present, researchers have successfully established several models of neurological diseases using tree shrews, including Alzheimer's disease (Li et al., 2024, Yang et al., 2022), Parkinson's disease, drug addiction, and ischemic stroke (Bo et al., 2024, Che et al., 2021, Li et al., 2024). However, there is still a lack of ideal TBI tree shrew models. In this study, we established a TBI tree shrew model and evaluated its stability from behavioral, imaging, and morphological perspectives, aiming to provide a potential bridge for translational medical research on TBI from rodents to humans.

## Materials and methods

### Animals

Adult healthy female tree shrews (6–7 months old, weighing approximately 110–250 g) were obtained from the Animal Experiment Center of Kunming Medical University (Kunming, China). The animals were housed under controlled conditions with a temperature of 20–25°C, relative humidity of 50–70 %, and a 12-hour light/dark cycle. Standard laboratory chow and water were provided *ad libitum*. All experimental procedures involving animals were performed in accordance with the Guide for the Care and Use of Laboratory Animals and approved by the Animal Ethics Committee of Kunming Medical University (Approval No.: YNSJ-SC-202412–012, License for Use: SCXK(Dian)K2020–0004).

### TBI model

**Fig. 1:**
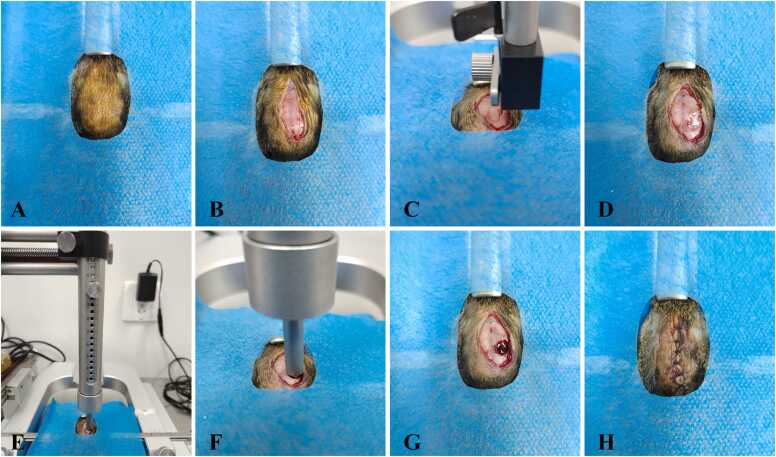
The preparation process of the TBI tree shrew model. A: Surgical skin preparation and disinfection. B: Cut open the scalp. C: Use the stereotactic instrument to determine the damaged area. D: Expose the dura mater. E and F: Craniocerebral injury was caused by using the Marmarou weight-drop impact method. G: General observation after craniocerebral injury surgery. H: Suture the incision and disinfection.

The Marmarou weight-drop impact method was used to establish a TBI model in tree shrews (Fig. 1). Briefly, tree shrews were anesthetized with sodium pentobarbital (40 mg/kg) and fixed on the stereotactic instrument. After disinfection, a midline scalp incision was made along the sagittal suture to expose the anterior fontanelle, coronal suture, sagittal suture, and occipital bone. A 5-mm diameter bone window was created in the right parietal bone (8 mm posterior to the anterior fontanelle and 4 mm lateral to the sagittal suture) using a dental drill. A brain contusion was induced by dropping a 60 g weight from a height of 10 cm onto a plastic plate placed over the dura mater. Following the impact, stop the bleeding, disinfect and suture the scalp. Sham-operated animals underwent the same surgical procedure without impact injury.

### Experimental design

Twelve adult female tree shrews were randomly divided into two groups: the Sham group and the TBI group. On the first and seventh days after the establishment of the TBI model, the autonomous movement ability of tree shrews was evaluated using the open field experimental device, subsequently, magnetic resonance imaging (MRI) scans were conducted. Finally, brain tissue sections of six experimental animals in each group were taken for HE staining and immunofluorescence staining. Plasma and brain tissues of six animals in each group were collected for ELISA and Western blot detections respectively.

### Open-field test

The autonomous movement ability of tree shrews was evaluated using an open field experimental device (equipped with the Super Maze+ behavioral analysis system, Shanghai Xinxin Information Technology Co., Ltd.). This test method was primarily based on the protocol described by Zhang et al (Zhang et al., 2022), with key modifications to accommodate the agile and stress-sensitive nature of tree shrews:

Apparatus Modification for Escape Prevention: An extra 10 cm-high transparent acrylic wall was added around the opening of the open-field box (length×width×height=80 cm×80 cm×50 cm, inner wall: black material) to prevent tree shrews from jumping out during the experiment. The wall was evenly perforated with 20 ventilation holes (diameter=1 cm, spacing=5 cm) to ensure air circulation and avoid animal overheating.

Standardized Test Environment and Procedure: The test was conducted in a sound-attenuated (noise <40 dB) and dimly lit room (illuminance=50 lux, warm yellow LED lights) to minimize external disturbances. Tree shrews were gently placed in the center of the arena by an experienced experimenter (using soft gloves to avoid skin scratches) to reduce handling-induced stress. Each test lasted strictly for 10 min to ensure data consistency, and movement trajectories of tree shrews were recorded continuously by the Super Maze+ system.

Odor Control Protocol: After each tree shrew completed the test, the arena was thoroughly cleaned with 75 % ethanol to eliminate residual odor cues, then dried naturally before next test to avoid odor interference on subsequent animals’ behavior.

### Magnetic resonance imaging

On the 7th day after modeling, tree shrews were anesthetized with isoflurane (3 % induction, 1.5 % maintenance) to ensure stable physiological status during scanning. A 0.3 T small-animal MRI scanner (PANION model, Meisi Medical Technology Co., Ltd.) was used to acquire T2-weighted images (T2WI) of the brain. Scanning parameters were set as follows: repetition time/echo time (TR/TE) = 1700/86 ms, slice thickness = 3 mm, and matrix size = 160 × 160. Six tree shrews in each group were included in MRI scans to ensure the comprehensiveness of imaging data.

### Hematoxylin-eosin (HE) staining

To assess neuronal damage, HE staining was performed using a commercial kit (Biosharp, Shanghai, China) following the manufacturer’s instructions. Stained sections were observed under a light microscope (YiJingTongGuang Technology, CX23LEDRFS1C, Guangzhou).

### Immunofluorescence staining

The 4-μm thick paraffin sections were deparaffinized, rehydrated, and subjected to antigen retrieval. Sections were blocked with 5 % normal goat serum and 0.3 % Triton X-100 for 3 h and incubated overnight at 4℃ with primary antibodies. The primary antibodies used were as follows: anti-CD86 monoclonal antibody (Proteintech, Cat# 13395–1-AP) diluted at 1:200, and anti-CD206 monoclonal antibody (Proteintech, Cat# 18704–1-AP) diluted at 1:200. The next day, the sections were washed with PBST and incubated with fluorescent secondary antibody (DyLight 488-conjugated Goat Anti-Rabbit IgG, Abbkine, Cat# A23220) diluted at 1:3000 at room temperature in the dark for 3 h. After washing with PBS, nuclei were stained with 4′,6-diamidino-2-phenylindole (DAPI) (C1002, Beyotime). Finally, sections were mounted, and images were captured using a fluorescence inverted microscope (Zeiss, Oberkochen, Germany).

### Enzyme-linked immunosorbent assay (ELISA)

Brain tissue samples (50 mg) were homogenized in 0.45 mL of pre-cooled PBS with two protein grinding beads at 70 Hz for 60 s, repeated twice. The homogenates underwent two freeze-thaw cycles at −80°C, followed by centrifugation at 12,000 g for 10 min at 4°C. The supernatant was collected and stored at −80°C for analysis. Plasma levels of TNF-α (ERC102a, NeoBioscience Technology Co., Ltd., Shenzhen, China), IL-6 (ERC003, NeoBioscience Technology Co., Ltd., Shenzhen, China), IL-10 (ERC004, NeoBioscience Technology Co., Ltd., Shenzhen, China), and IL-13 (JL20877, Jianglai Biotechnology Co., Ltd., Shanghai, China), as well as brain tissue levels of TNF-α, IL-6, IL-10, IL-13, C3 and S100a10, were measured using ELISA kits according to the manufacturer’s instructions.

### Western blot analysis

Tissue samples stored at −80°C were homogenized in RIPA buffer (R0010, Solarbio) to extract total protein. Protein concentration was determined using a BCA protein assay kit. Total protein was separated by 10 % SDS-PAGE and transferred onto a polyvinylidene fluoride (PVDF) membrane (Millipore, Billerica, USA). Membranes were blocked with 5 % BSA at room temperature for 2 h and incubated overnight at 4°C with primary antibodies: Serping1 (66882–1-Ig, Proteintech, 1:1000), Ptx3 (13797–1-AP, Proteintech, 1:1000), and rabbit anti-β-actin (20536–1-AP, Proteintech, 1:5000). The following day, membranes were incubated with secondary antibodies at room temperature for 60 min, followed by development, imaging, and data storage.

### Statistical analysis

All the data were analyzed with GraphPad Prism 10.1.2 (GraphPad Software, La Jolla, USA). Data were expressed as mean ± standard error of the mean (SEM). For comparisons between two groups, independent-sample *t*-tests were performed if the data followed a normal distribution; otherwise, nonparametric tests were applied. A p-value < 0.05 was considered statistically significant.

## Results

### The TBI model of tree shrew was successfully constructed in this study

**Fig. 2:**
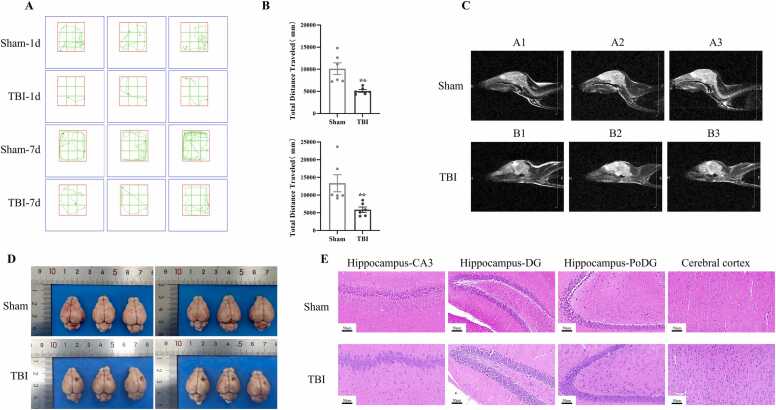
The TBI model of tree shrew was successfully constructed. A: The motion trajectory diagrams of the open field test on the 1st and 7th days after TBI surgery. B: The total distance traveled in the open field experiment of the two experimental groups on the 1st and 7th days after TBI surgery was statistically compared (n = 6/group). C: Magnetic resonance imaging of tree shrew brains in the Sham group and the TBI group (n = 6/group). D: Comparison of gross brain tissue specimens of tree shrews in the Sham group and the TBI group (n = 6/group). E: HE staining images of brain tissue sections of tree shrews in the Sham group and the TBI group (n = 6/group). ^**^: p＜0.01 *v.s.* Sham.

After TBI model establishment, the open-field test was conducted to evaluate the motor capacity of tree shrews. Results showed that compared with the Sham group, the total motor distance of tree shrews in the TBI group was significantly reduced on post-injury day 1 (a 50 % decrease, p < 0.01) and day 7 (a 39 % decrease, p < 0.01), indicating that TBI causes sustained impairment of motor ability (Figs. 2A, 2B). Magnetic Resonance Imaging (MRI) results revealed that the brain parenchyma of the Sham group exhibited uniform signals with no abnormal density shadows. In contrast, the TBI group showed patchy high-density shadows (approximately 4 mm in diameter) in the parietal brain parenchyma, mixed with irregular patchy hemorrhage signals—indicating acute intracerebral hemorrhage—accompanied by low-density edema bands in the surrounding brain tissue (Fig. 2C). These MRI features are consistent with traumatic cerebral edema with space-occupying effect and are highly associated with behavioral dysfunction.

Gross observation of tree shrew brain tissues on post-injury day 7 showed that the Sham group had smooth brain tissue surfaces, uniformly light pink color, clear gyrus and sulcus structures, regular vascular distribution, and no hemorrhage or edema. In the TBI group, distinct dark red hemorrhagic lesions (3–5 mm in diameter) with surrounding grayish areas were observed; local gyri were flattened and structurally damaged, and blood vessels in the injured area were tortuous, congested, or ruptured (Fig. 2D).

Hematoxylin-Eosin (HE) staining results demonstrated that in the Sham group, hippocampal cells were arranged regularly, neurons had intact morphology with large, round nuclei, and no pathological changes (e.g., inflammation, edema, hemorrhage, or necrosis) were observed. Cortical neurons were arranged in layers, with slight differences in cell density and morphology among layers. In the TBI group, some hippocampal neurons showed morphological abnormalities and nuclear pyknosis, accompanied by glial cell proliferation; cortical neurons were disorganized, with disrupted layered structures in some regions and morphological abnormalities of neurons (Fig. 2E).

### The expression levels of inflammatory factors in brain tissue and plasma of tree shrew were changed after TBI modeling

**Fig. 3:**
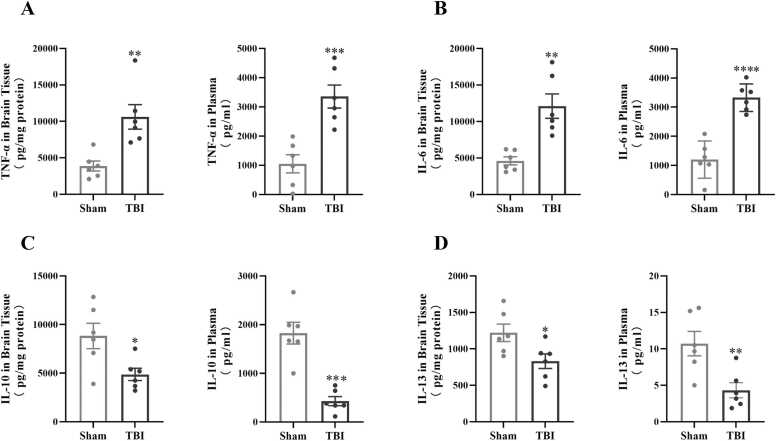
The expression levels of inflammatory factors in brain tissue and plasma of tree shrew were changed after TBI modeling. A-D: Comparisons of TNF-α, IL-6, IL-10 and IL-13 contents in tree shrew brain tissue and plasma 7 days after TBI respectively (n = 6/group). *: p＜0.05 *v.s.* Sham, ^**^: p＜0.01 *v.s.* Sham, ^***^: p＜0.001 *v.s.* Sham, ^****^: p＜0.0001 *v.s.* Sham.

In order to identify the occurrence of neuroinflammation after TBI, the expression levels of pro-inflammatory factors (TNF-α, IL-6) and anti-inflammatory factors (IL-10, IL-13) in tree shrew plasma and injured brain tissue were detected by ELISA respectively. The results showed that compared with Sham group, the levels of pro-inflammatory factors TNF-α and IL-6 in brain tissue and plasma of tree shrews in TBI group were significantly increased (Figs. 3A, 3B), while the anti-inflammatory factors IL-10 and IL-13 decreased significantly (Figs. 3C, 3D).

### After TBI, microglia and astrocytes showed "biphasic activation" phenomenon

**Fig. 4:**
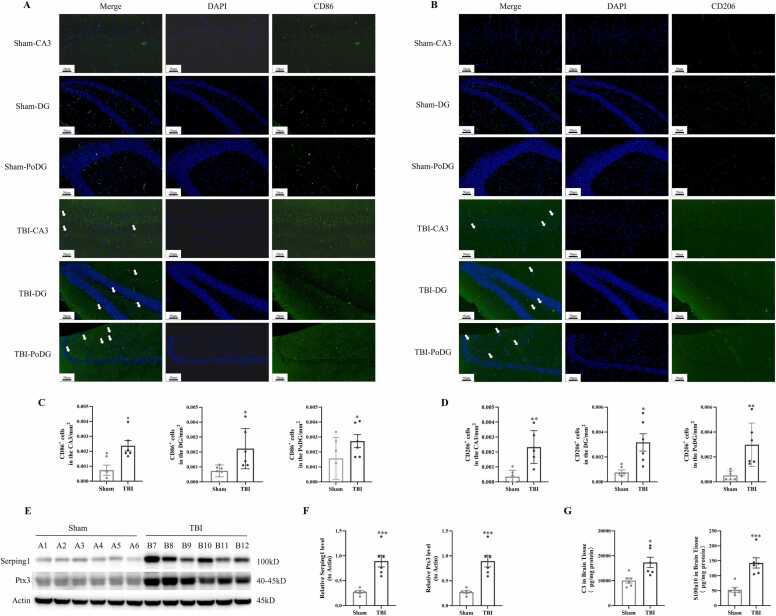
After TBI, microglia and astrocytes showed "biphasic activation" phenomenon. A-B: Immunofluorescence staining showed the expression of CD86 and CD206 positive microglia in the hippocampal CA3, DG and PoDG regions of tree shrews brain tissues in each experimental group (n = 6/group). C-D: Semi-quantitative statistical analysis of CD86 and CD206 fluorescence signals in the CA3 region, DG region and PoDG region of the hippocampus in the TBI group and the Sham group of tree shrews respectively. E: Western Blot was used to detect the expression level of Serping1 and Ptx3 proteins in the brain tissues of tree shrews in each experimental group (n = 6/group). F: Semi-quantitative statistical results of the expression levels of Serping1 and Ptx3 proteins in the brain tissues of tree shrews in each experimental group. G: ELISA was used to detect the expression levels of C3 and S100a10 in the brain tissues of tree shrews in each experimental group (n = 6/group). The white arrow indicates CD86 and CD206 positive microglial cells. *: p＜0.05 *v.s.* Sham, ^**^: p＜0.01 *v.s.* Sham, ^***^: p＜0.001 *v.s.* Sham.

Immunofluorescence staining results revealed that, compared with the Sham group, the fluorescence signals of the M1 microglia phenotype marker CD86 in the hippocampal CA3, DG, and PoDG regions of the TBI group were significantly enhanced at 7 days post-TBI (Figs. 4A, 4C). Similarly, the fluorescence signals of the M2 phenotype marker CD206 in the same hippocampal regions of the TBI group were also notably increased compared to the Sham group (Figs. 4B, 4D). Western Blotting analysis demonstrated that, relative to the Sham group, the protein expression levels of the astrocyte A1 phenotype marker Serping1 and A2 phenotype marker Ptx3 in the TBI group were significantly up-regulated (Figs. 4E, 4F). Additionally, ELISA results showed that the expression levels of C3 and S100a10 in the brain tissue of the TBI group were significantly higher than those in the Sham group (Fig. 4G).

## Discussion

Reliable animal models are the basis for exploring the pathological mechanisms and molecular events of secondary brain injuries (such as oxidative stress, neuroinflammation, and programmed cell death), and are helpful in finding molecular targets to alleviate secondary brain injury and improve brain function (Alam et al., 2020). In this study, we successfully constructed a TBI tree shrew model. Behavioral tests showed that the motor ability and neurological function of tree shrews significantly decreased after TBI, which was highly consistent with the clinical phenotype of human TBI. Imaging features suggested that in the TBI group, there were signs such as acute hemorrhage and edema in the brain tissue around the injury area of tree shrews, which was consistent with the occurrence of edema in the brain tissue and space-occupying effect after trauma (Packer et al., 2023). The brain tissue specimens of tree shrews in the TBI group showed hemorrhage foci, structural damage and vascular destruction in the injured area，HE staining showed that the neurons in the injured area were disordered in arrangement, abnormal in morphology, nuclear condensation and glial cell proliferation. These above research results confirmed that after TBI, tree shrews exhibited typical trauma characteristics of "motor behavior dysfunction-imaging abnormalities-histopathological damage", indicating the successful construction of the TBI tree shrew model.

After TBI, the neuroinflammatory response is one of the factors causing secondary brain injury. Our study found that after TBI, in the brain tissue and plasma of tree shrews, the levels of inflammatory factors TNF-α and IL-6 significantly increased, while the expression levels of anti-inflammatory factors IL-10 and IL-13 significantly decreased. Both the injured local brain tissue and the whole body show a bidirectional imbalance feature of significantly up-regulating the expression of pro-inflammatory factors and significantly down-regulating the expression of anti-inflammatory factors, suggesting the cascade activation of neuroinflammation. The continuous release of pro-inflammatory factors may directly mediate neuronal injury and the disruption of the blood-brain barrier (Jacquens et al., 2022). Insufficient secretion of anti-inflammatory factors leads to the failure of negative feedback regulation of inflammation, further intensifying the vicious cycle of the inflammatory microenvironment and ultimately resulting in irreversible neurological function damage (Sulimai & Lominadze, 2020).

To preliminarily clarify the mechanism of neuroinflammation in the TBI tree shrew model, this study observed the polarization status of microglia and astrocytes after TBI. It was found that both CD86 and CD206 positive microglia in the hippocampal region of the TBI tree shrew brain tissue increased, suggesting biphasic activation of microglia, that is, the synchronous activation of anti-inflammatory/repair phenotypes. On the one hand, the significant increase in the M1 phenotype of microglia in brain tissue indicates an intensified pro-inflammatory response, which may aggravate secondary injury by releasing neurotoxic factors (such as TNF-α, IL-6) (Wangler & Godbout, 2023). On the other hand, the synchronous upregulation of the M2 phenotype in brain tissue suggests the initiation of the endogenous repair mechanism. However, the expressions of anti-inflammatory factors IL-10 and IL-13 in peripheral blood were significantly downregulated after TBI, suggesting that the repair function of M2-type microglia may be limited. This "decoupling" phenomenon of the synchronous activation of the M1/M2 phenotype of microglia in brain tissue and the expression of anti-inflammatory factors in peripheral blood indicates that the endogenous repair mechanism after TBI fails to effectively antagonize the neuroinflammatory response, resulting in an imbalance of pro-inflammatory/anti-inflammatory homeostasis (Li et al., 2022, Liu et al., 2025). The neurotoxic factors (such as TNF-α and IL-6) continuously released by the activation of M1-type microglia may further inhibit the anti-inflammatory function of M2-type cells, forming a vicious cycle and ultimately exacerbating neuronal injury and neurological dysfunction (Willis et al., 2020, Wu et al., 2024). These results suggest that inducing M2 polarization of microglia may become a potential therapeutic target for TBI.

Meanwhile, the expressions of astrocyte type A1 marker Serping1 and complement C3 in the brain tissue of the TBI group were significantly higher than those in the Sham group, and the expressions of type A2 markers Ptx3 and S100a10 were also significantly increased, showing the characteristic of A1/A2 phenotypic imbalance. As an inflammatory regulatory protein, the upregulation of Serping1 expression may exacerbate neuroinflammation and the disruption of the blood-brain barrier by inhibiting the activation of plasminogen (Sulimai and Lominadze, 2020, Zhao et al., 2017). The abnormal activation of the C3 complement system can mediate excessive synaptic pruning (Jamjoom et al., 2021), neuronal apoptosis and glial scar formation (Muñoz-Ballester & Robel, 2023). Although the expressions of type A2 markers Ptx3 and S100a10 in astrocytes in the TBI group were also significantly increased, the expression levels of anti-inflammatory factors IL-10 and IL-13 in peripheral blood decreased, suggesting that the repair function of type A2 cells was limited. As an acute response protein, the upregulation of Ptx3 may reflect the early compensation of tissue injury repair, but it cannot effectively antagonize the inflammatory injury dominated by type A1 astrocytes (Izzy et al., 2025, Yuan and Wu, 2022). S100a10 is associated with cellular stress and leakage of the blood-brain barrier, its overexpression may exacerbate oxidative stress and neuronal metabolic disorders (Qin et al., 2025), ultimately leads to neurological deficits in patients with TBI.

In conclusion, this paper successfully constructed the TBI tree shrew model and confirmed that the polarization imbalance of microglia and astrocytes may form a neurotoxic microenvironment, ultimately causing brain tissue damage.

## Funding information

10.13039/501100001809National Natural Science Foundation of China, Grant/Award Number: 82260384; Foundation Research Project of the Science and Technology Department of Yunnan Province, Grant/Award Number: 202501AY070001–264; Key Research and Development Program of the Health Commission of Yunnan Province, Grant/Award Number: 202403AC100010.

## CRediT authorship contribution statement

**Xiaolina Zhang:** Methodology. **Jie Zhang:** Software. **Jiayu Zhang:** Visualization. **Yongjie Huang:** Validation. **Linbo Wang:** Data curation. **Linyi Chen:** Investigation. **Shengxiong Hong:** Investigation. **Jinglin Li:** Writing – review & editing, Methodology, Funding acquisition. **Haiying Wu:** Writing – review & editing, Funding acquisition. **Li Yang:** Writing – original draft. **Wen Yu:** Writing – original draft.

## Declaration of Competing Interest

The authors declare that they have no known competing financial interests or personal relationships that could have appeared to influence the work reported in this paper.
